# LM-Merger: a workflow for merging logical models with an application to gene regulatory network models

**DOI:** 10.1186/s12859-025-06212-2

**Published:** 2025-07-15

**Authors:** Luna Xingyu Li, Boris Aguilar, John Gennari, Guangrong Qin

**Affiliations:** 1 Institute for Systems Biology, Seattle, WA 98109 USA; 2 Department of Biomedical Informatics and Medical Education, University of Washington, Seattle, WA 98195 USA

**Keywords:** Gene regulatory networks, Logical models, Model integration, Acute myeloid leukemia, Systems biology

## Abstract

**Background:**

Gene regulatory network (GRN) models provide mechanistic understanding of genetic interactions that regulate gene expression and, consequently, influence cellular behavior. Dysregulated gene expression plays a critical role in disease progression and treatment response, making GRN models a promising tool for precision medicine. While researchers have built many models to describe specific subsets of gene interactions, more comprehensive models that cover a broader range of genes are challenging to build. This necessitates the development of approaches for improving the models through model merging.

**Results:**

We present LM-Merger, a workflow for semi-automatically merging logical GRN models. The workflow consists of five main steps: (a) model identification, (b) model standardization and annotation, (c) model verification, (d) model merging, and (e) model evaluation. We demonstrate the feasibility and benefit of this workflow with two pairs of published models pertaining to acute myeloid leukemia (AML). The integrated models were able to retain the predictive accuracy of the original models, while expanding coverage of the biological system. Notably, when applied to a new dataset, the integrated models outperformed the individual models in predicting patient response.

**Conclusions:**

This study highlights the potential of logical model merging to advance systems biology research and our understanding of complex diseases. By enabling the construction of more comprehensive models, LM-Merger facilitates deeper insights into disease mechanisms and enhances predictive modeling for precision medicine applications.

**Clinical trial number:**

Not applicable.

**Supplementary Information:**

The online version contains supplementary material available at 10.1186/s12859-025-06212-2.

## Introduction

Gene regulatory networks (GRN) are fundamental to a wide range of biological processes, including cell differentiation, signaling transduction, and cell cycle in both normal and disease states [1]. GRNs provide virtual representations of biological systems, which can then be used for simulation of the dynamics of the physical systems. With these, we can make predictions about disease progression or drug response in a personalized manner, taking into account an individual's genetic profiles for genes included in the model. GRNs can be modeled using various approaches, including logical models, ordinary differential equations (ODEs), and piecewise linear differential equation models [2]. Among them, logical models are appealing due to their simplicity and versatility, especially in cases where kinetic parameters are unavailable [3, 4]. Logical GRN models have been successfully applied to study a wide range of diseases, including breast cancer [5], pancreatic cancer [6], and acute myeloid leukemia (AML) [7].

Building GRN models requires extensive domain expertise. Due to the limitation of knowledge from individual research teams, and the focus of different studies, existing published models are usually focused on one process or theme, with limited coverage of genes and processes for a more systematic study. Different models also use different representations for the nodes and relationships, which further complicates any efforts at model integration. To address this, the community has emphasized the need for systematic methods to integrate previously developed GRNs (e.g., in [2, 5]). Such integrated models are essential for illustrating complex biological phenomena and understanding the intricate regulatory mechanisms that drive cellular behavior [8, 9].

One integration approach is to develop modular models, where distinct biological processes are modeled independently and combined during simulation via variable transformations and synchronization [10]. While modular models hold promise, they face challenges in standardization and harmonization across models, which remain labor-intensive and error-prone. Furthermore, modular models often fail to preserve key network features, such as feedback loops and dynamic interactions among components, as their simulations are kept independent. Directly integrating individual models overcomes these limitations by preserving network structures and enabling continuous information exchange. Tools like RegNetwork [11] and NDEx [12] provide solutions by leveraging shared regulatory components from multiple sources, but they primarily focus on visualization and storage rather than computational analysis. Whole-cell models (WCM) represent another ambitious effort to simulate entire cellular processes by integrating diverse biological networks [13]. Yet, WCMs are currently limited to a few organisms such as *M. genitalium* [14] and *E. coli* [15], and integrating heterogeneous data remains a major challenge [16]. Notably, most existing tools and workflows focus on ODE and rule-based models, leaving logical models underserved.

In this study, we developed a workflow for merging logical GRNs models (LM-Merger), and demonstrated its effectiveness in enhancing the understanding and coverage of GRNs. We applied this workflow to AML, a highly aggressive hematopoietic malignancy characterized by the accumulation of somatic genetic alterations that disrupt normal cell behavior [17–22]. AML cells from different patients often carry diverse mutations [19, 20, 22, 23], which are associated with varied drug responses [22]. Numerous GRN models have been developed to investigate different aspects of AML, including hematopoietic stem cells regulation [24], potential drug responses [25], and predictions of clinical outcomes [7]. By combining two pairs of published AML models, we created larger, more comprehensive models that retained the behaviors of original models while offering broader insights. These merged models are better suited for predicting gene expression and clinical outcomes in AML patients. Furthermore, this workflow can be applied to other application areas where a systematic model-merging approach can be beneficial. At present, LM-Merger is implemented for Boolean (binary-state) logical models; support for other logic formalisms will require additional consideration and is discussed in Supplementary methods.

## The logic model merging workflow

**Fig. 1:**
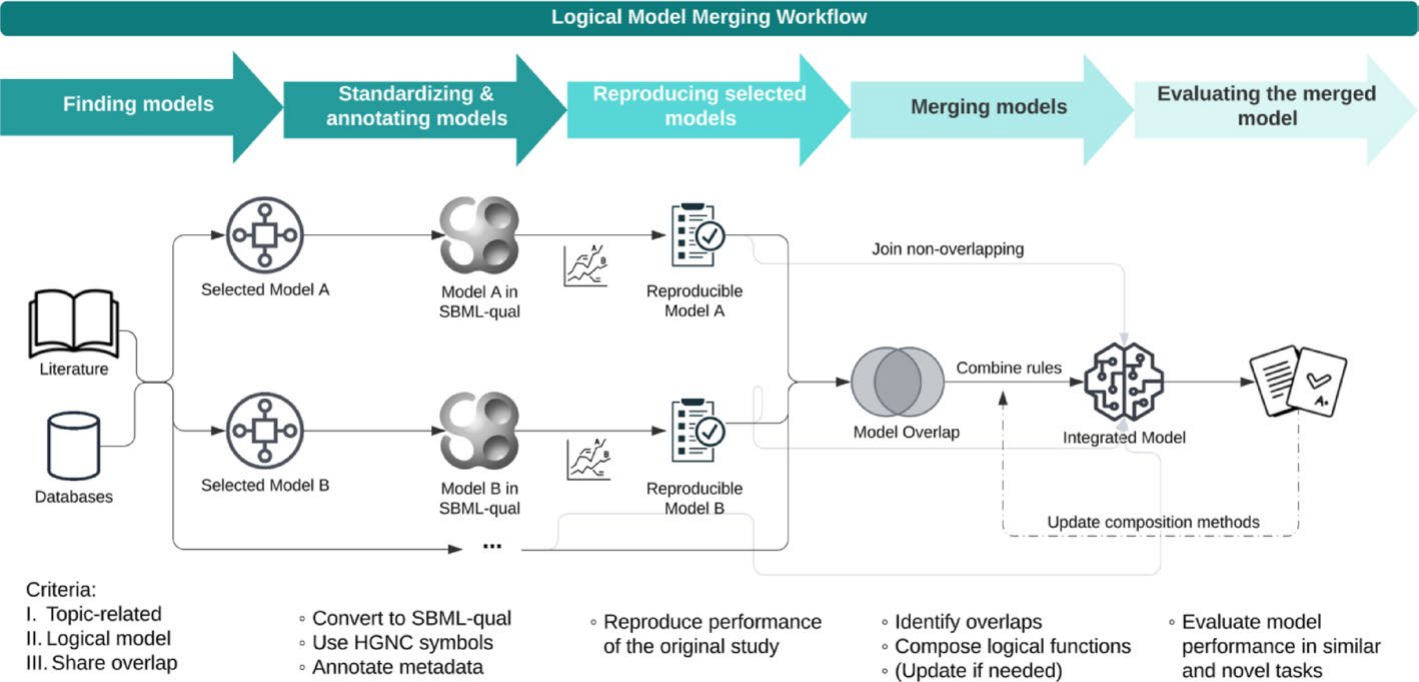
Overview of the logical model workflow. The flowchart illustrates the step-by-step process for merging logical models. The workflow begins with collecting eligible models from literature and databases. These models are then standardized into the SBML-qual format with added annotations. Reproducibility is verified for each model before proceeding to the merging step. The merging steps include composition of rules for overlapping nodes and non-overlapping components are directly integrated. Finally, the integrated mode is evaluated on tasks similar to their original studies and novel tasks of interest, ensuring its accuracy and applicability.

The LM-Merger workflow integrates logical models from various sources into a unified representation to provide a more comprehensive view of biological mechanisms. It includes five main steps: (1) Finding models, (2) Standardizing and annotating models, (3) Reproducing selected models, (4) Merging models, and (5) Evaluating the merged model (Fig. 1A). While this section focuses on describing the workflow, details on its implementation in specific use cases are provided in the Supplementary methods.

### Finding models

The initial phase of integrating logical models involves identifying candidate models. This typically starts with existing data like publications and repositories relevant to the biological system under investigation. Researchers often look into large repositories of logical models, such as the Cell Collective [26], the GINsim repository [27] and BioDiVinE [28], to find logical models of interest. Pathway databases like KEGG [29], Reactome [30], and causal interactions such as SIGNOR [31] can also be useful resources, although they only provide interaction graphs and need to be translated into logical relationships afterwards. A simple approach to this translation is to use the ‘Inhibitor Wins’ combination method, where inhibitory interactions dominate over activatory ones (See the following section).

After collecting logical models and building a library of candidate models, each model should be carefully reviewed to assess its relevance based on the topic, components involved, and the knowledge source. The decision should be primarily based on the purposes of the models and their biological relevance. Another important consideration is the level of overlap, as we aim for the models to complement each other by expanding the coverage of nodes while sharing key regulators. The amount of overlap may depend on the biological context and use case for the models. For example, to integrate models of a specific disease pathway with other models for drug response prediction, we would consider models that share at least some critical pathway mediators, so that the drug effect can be passed on to the phenotypes of the pathway.

### Standardizing and annotating models

Reproducibility, a long-standing concern of the scientific community, can be significantly improved through the use of standards, annotations and repositories [32]. The accurate and appropriate description of both the model itself and its components is also essential for model composition. As proposed by the curation and annotation of logical models (CALM) initiative [33], we use the Systems Biology Markup Language Qualitative Models (SBML-qual) format [34, 35] to encode the selected models.

The community often refers to the HUGO Gene Nomenclature Committee (HGNC) guidelines for naming human genes [36]. Additionally, the Vertebrate Gene Nomenclature Committee encourages the use of the human orthologs for other vertebrate species, such as for mouse and rat. For candidate models that originate from other species, an orthology-mapping step is required. We recommend first reconciling each node to its human HGNC symbol and flagging nodes whose regulatory logic is known to diverge between species, which would require additional expert curation. In this workflow, we mapped each gene in the models to HGNC gene ID and annotated them with HGNC approved symbols. We implemented a semi-automated approach for this step, where standardized gene names can be queried through the HGNC REST API. After manual curation, the results can then be used to update the SBML-qual model. Following the International Protein Nomenclature Guidelines [37], we use the same abbreviations for proteins as their corresponding genes. Special attention should be given to fusion proteins and complexes, for which standard nomenclature may not exist. Metadata including original sources of the models should also be included in the annotation. We propose linking each component in the models to online resources, and annotating the supporting evidence of the edges, e.g., the experimental method used to determine the interaction, and the source of the data. This ensures that the models are transparent and compatible for integration.

### Reproducing selected models

As models become more complex, the reproducing task becomes more difficult [38, 39]. Before merging, each model is verified by replicating its published results under the same conditions. The results are then compared to the published performance to ensure that models are correctly implemented.

We used the CoLoMoTo Interactive Notebook in this study, which provides a unified environment for performing analyses and validating behavior of logical models [40]. The advantage of using this tool is to ensure the reproducibility of results by using the same computational environment for different models.

### Composing models

Logical models represent GRNs as networks where nodes are genes or proteins, and edges represent regulatory interactions between them. The state of each node is determined by a logical rule $f$, which describes how the states of its regulators influence its activation. These logical functions are the core of the network and define its dynamic behavior. Using a combination of logical operations, we can then describe the updating schema for a gene or any biological molecule [41]. We restrict the logical operators to logical product (AND, represented by the symbol ‘&’), logical sum (OR, symbol ‘|’), and logical negation (NOT, symbol ‘!’).

Suppose there are *n* logical models ${M}_{j=1}^{n}$ to be integrated, it is necessary to address both overlapping and non-overlapping components within them. For overlapping nodes that are shared by the models, the logical rules ${f}_{i}$ governing their behavior for each model $i$ needs to be merged. We propose three deterministic methods for this merging process, each reflecting a different biological rationale. Here, ${f}_{i}^{merged}$ represents the function of the ${i}^{th}$ nodes after merging, using the functions from their original models ${f}_{i}^{{M}_{j}}$:

1. **OR Combination**: $$\:{f}_{i}^{OR}={\bigvee\:}_{j=1}^{n}{f}_{i}^{{M}_{j}}$$

This method combines the logical rules from the individual models using the logical OR operator. If either of the rules from the models predicts the activation of a node, the combined rule will also predict activation. This approach ensures that the integrated model captures all possible activation scenarios, providing a more inclusive representation of the regulatory network.

2. **AND Combination**: $$\:{f}_{i}^{AND}={\bigwedge\:}_{j=1}^{n}{f}_{i}^{{M}_{j}}$$

In contrast, the AND combination method uses the logical AND operator to merge the rules. Here, a node will only be activated in the integrated model if both original models predict its activation. This method is more stringent, ensuring that only consistent activation predictions are retained, which can reduce false positives and emphasize strong, corroborated regulatory interactions.

3. **Inhibitor Wins Combination**:
  $$ f_{i} ^{{IW}} = \left\{ {\begin{array}{*{20}l} 0 \hfill & {i{\text{f}}\;\exists j\;{\text{such that}}\;{\text{ }}f_{i} ^{{M_{j} }} \;{\text{contains any active inhibitor}}} \hfill \\ { \vee ^{n} _{{j = 1}} f_{i} ^{{M_{j} }} } \hfill & {{\text{otherwise}}} \hfill \\ \end{array} } \right. $$

This method prioritizes inhibitory interactions. If any edge in the original models represents an inhibitory relationship, this inhibition will dominate in the merged model, leading to the node being turned off or having a negative impact on its status. This approach reflects the biological reality where inhibitory signals often have a strong regulatory effect, such as in the suppression of oncogenes or other critical pathways [7, 42].

Given that gene regulation can be viewed as the interplay of transcription factors (TFs) and TF-binding sites of mRNA to govern expression levels of mRNA and their resulted proteins [43], it is intrinsically related to enhancer function and logic [44, 45]. Here, the three merging strategies offer complementary interpretations: the “OR” combination reflects the flexibility and robustness seen in enhancer integration, where multiple transcription factors (TFs) can independently activate a gene. The “Inhibitor Wins” approach represents the biological reality where repressive signals prevent inappropriate gene expression. This is supported by studies on transcriptional repression, where inhibitors can override activatory signals [46]. The “AND” method enforces the strictest criteria, and is analogous to the cooperative binding of TFs for fine-tuned gene expression control. Researchers can select the most appropriate merging method based on the goals of the study, whether emphasizing inclusivity, stringency, or regulatory dominance. (See Supplementary methods for an example.) Although our examples in this manuscript apply a single rule-combination uniformly, LM-Merger offers a method to tailor strategies node-by-node by a customization function. This lets users, for example, apply ‘Inhibitor Wins’ to tumour-suppressor genes while using ‘OR’ elsewhere.

### Evaluating the merged model

Typically, evaluation of the logical models includes both dynamic and static analysis [47]. Attractors or stable states can be associated with cellular phenotypes and represent the long-term behavior of the system. Simulation of the models can provide insights on possible trajectories, and can be used to predict response of the system to perturbations such as gene mutations. However, the specific methods should be determined by the user and the biological goal of the models. We provide some helper functions in the workflow for these tasks.

The aim of evaluation should be twofold: (1) to ensure that the integration maintains the functionality or performance of the original models, and (2) to demonstrate or explore new capabilities on new datasets. The first step is to verify if the merged model behaves similarly and retains the predictive accuracy of the individual models. Second, we test the merged model on new tasks to assess its robustness on different biological scenarios, such as predicting gene expression under new conditions or the impact of novel mutations. Finally, by comparing results from different integration strategies, we determine which approach best captures the biological processes and provides the most accurate predictions. We can then choose to employ the integration strategy that offers the highest accuracy and predictive power while aligning with the biological context of the study.

## Results: use-case demonstration with AML models

We applied the workflow on two pairs of published logical models on AML to demonstrate the benefits of model integration for complex biological processes. Per the workflow, we first carried out a broad literature search to identify relevant logical models related to gene regulation in AML. The supplementary materials (Tables S1 and S2) provide literature search details. Because AML originates from hematopoietic stem cells (HSC) that have undergone malignant transformation [48], our search included models that describe HSC behavior. From the retrieved models, we selected two pairs of models based on their overlap and biological relevance. The first pair uses mouse data and investigates HSC development; the second pair uses human data and investigates AML disease progression.

### Merge models to better simulate hematopoiesis

**Fig. 2:**
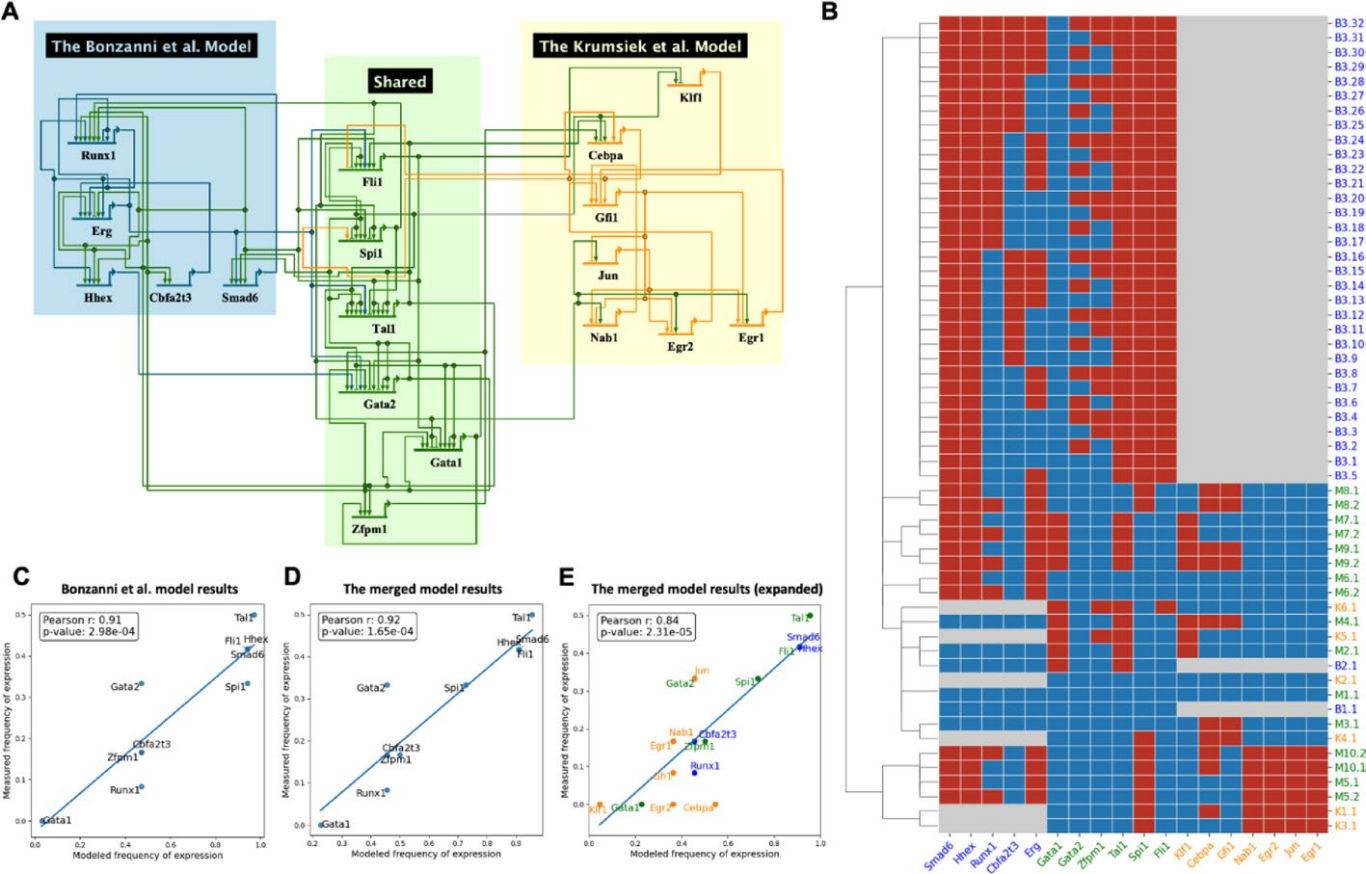
The merged model of Bonzanni et al. 2011 and Krumsiek et al. 2013 and its evaluation. **A** Visualization of the merged model using BioTapestry [71]. Lines indicate regulation relationships that point from the regulator to its targets, with arrowheads as activating and bars as repressing. Colors of the nodes and their downstream edges indicate which model they come from (or shared between models). **B** Steady states pattern of the merged model pair and individual models. Each row is a steady state of the Bonzanni et al. model (start with ‘B’), Krumsiek et al. model (start with ‘K’), or the **merged model** (Start with ‘M’). The color in the heatmap indicates that a gene is ON (red), or OFF (blue), or that the gene is not included in the model (Grey). (**C**–**E**) Correlation of measured frequency of expression (from experiments) with the modeled frequency of activation (from model simulation): **C** Results of the Bonzanni et al. model alone. **D** Results of the **merged model**. **E** Results of the **merged model** with an expanded coverage. 8 additional genes not covered in the Bonzanni et al. model are colored in orange. Results of the ‘OR’ model are shown, for other merged models results, see Figs. S1–S2.

The first model pair consists of Boolean models constructed by Bonzanni et al. [49] and Krumsiek et al. [50]. Both of these models describe crucial stages in hematopoietic differentiation, but with different emphases. The former model captures key regulatory genes of early HSCs and simulates the differentiation of stem cells into mature blood cells, including erythroids, monocytes, and granulocytes. The latter model focuses on a later stage of the disease, describing the transition of common myeloid progenitors into myeloid cells. The two models share 6 genes; see Fig. 2A. Importantly, both models include key AML-related genes absent in the other model; the Bonzanni model includes *Runx1* while the Krumsiek model includes *Cebpa*, both of which are involved in the pathogenesis of AML [51, 52]. Since AML can originate from various stages of this differentiation process, merging these models offers a more detailed explanation of the HSCs differentiation process and thus possibly a more comprehensive representation for AML patients with different genetic alterations. (Note that this model pair was tested using mouse data, therefore, we use a different capitalization naming convention.)

We merged the models using our three different integration strategies: ‘AND’, ‘OR’ and ‘Inhibitor Wins’, and performed steady state analysis using the asynchronous updating schema to identify both stable and cyclic attractors. Figure 2B shows the attractors for the simulation based on the ‘OR’ rule. The Krumsiek model has 6 attractor states, the Bonzanni model has 3 (including a large cyclical state, B3.x), and our merged model has 9. To evaluate the consistency and robustness of the merged model, the attractor patterns of both the individual models and the merged model are clustered based on the hamming distance. A common goal of the two models is to describe cellular behavior of HSCs. It has been reported that each of the individual models can generate states that are representative of the different cell types that can originate from HSCs [49, 50]. Our results demonstrated the reproducibility of the original papers with the same stable states, where states B2.1 and K5.1 represent erythroid cells. One interesting finding is that the merged model combines them in a single state M2.1, where the expression of *Hhex*, *Runx1* and *Erg* are inactive and *Gata1*, *Tal1* and *Klf1* are active. This has been demonstrated by microarray experiments of erythroid cells [53]. (See Fig. S2 for the comparison.) Additionally, in the Bonzanni paper, the authors claimed that an interconnected cyclic attractor of 32 states (B3, in Fig. 2B) represents a heterogeneous cell population of HSCs. In our merged model, we identified two attractor states (M3.1 and M3.2) that are clustered with the B3 state, featuring multiple genes active and *Gata1* consistently repressed. In contrast to the Bonzanni model, cyclic attractor M3.1 and M3.2 of the merged model do not show a variability in *Erg* and *Gata1*, suggesting their unique roles in HSC maintenance [54, 55].

To evaluate the model quantitatively, we compared the frequency of expression of genes in stable states with gene expression data from single-cell microarray experiments of HSCs similar to the previous study [56]. See Supplementary material for details. Results of the merged model show a strong correlation (Fig. 2D), comparable to the Bonzanni model alone (Fig. 2C). We then asked whether the merged model is predictive for an extended set of genes from the Krumsiek model, and obtained a correlation of 0.84 (Fig. 2E). Notably, many of the critical cancer related genes can only be modelled using the merged model, e.g., *Jun* and *Cebpa*, as colored in yellow. Another observation is that the performance of merged models using different approaches varies, with the ‘OR’ model having the highest correlation (Fig. S3). A possible reason is the heterogeneity of HSCs data, where multiple pathways may be activated simultaneously. This highlights the importance of choosing and evaluating approaches based on the context.

### Merge models to simulate AML disease progression

**Fig. 3:**
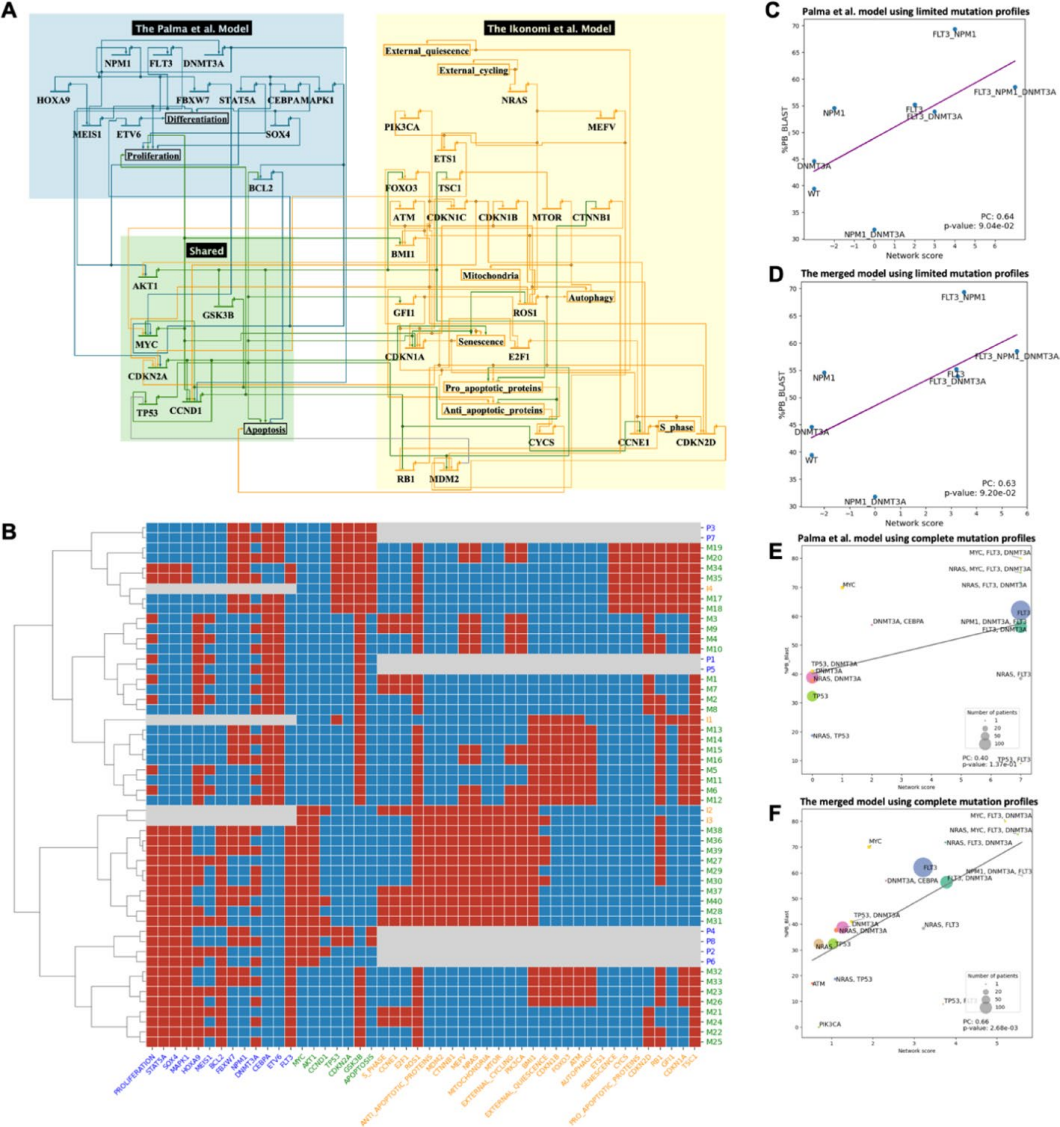
The merged model of Palma et al. 2021 and Ikonomi et al. 2020 and its evaluation. **A** Visualization of the merged model using BioTapestry [71]. **B** Steady states pattern of the **merged model** and individual models. **C**, **D** Correlation between the average blast percentage and network scores derived from the Palma et al. model (**C**) and from the **merged model** (**D**) for mutation status on FLT3, NPM1 and DNMT3A. **E**, **F** Correlation between blast percentage and network scores derived from the Palma et al. model for mutation status on all available genes (**E**) and from the **merged model** (**F**), which covers more genes. Size of node indicates number of patients for each mutation profile. Only scatterplot of the ‘AND’ model is shown, for other merged models results, see Fig. S4

Next, we evaluate a pair of models that focus on AML directly (Fig. 3A). The Palma et al. model [7] integrates patient-specific genomic data into a Boolean network to predict clinical outcomes in AML patients, focusing on key regulatory pathways and frequent mutations in AML. The Ikonomi et al. model [57] uses a Boolean network to model HSC maintenance and the regulation of *TP53* pathway in response to niche interactions. These models overlap in key regulatory pathways involved in hematopoiesis and AML. By merging the two models, we can gain a deeper understanding of how genetic mutations in AML impact both the differentiation of blood cells and their interactions with signals from the stem cell environment, providing insights into the disease’s progression and potential therapeutic targets.

As with the first pair, the merged model replicates the steady states of the original models (Fig. 3B). The Palma model has 8 attractor states, the Ikonomi model has 4, and our merged model has 40, many of which are combinations of states from individual attractors. One possible reason for the increased number of attractors in the merged model is that combining rules introduce novel feedback loops, thereby creating emergent attractors not reachable in either individual network. They could represent additional, biologically plausible gene-expression patterns (cell fates), and give researchers new testable hypotheses and a richer landscape for in-silico perturbation studies. In this model pair, each steady state from the individual models corresponds to several steady states in the merged model, which represents normal cellular function without perturbation. As an example, consider the attractors I1, from the Ikonomi model, and M13-M16, from the merged model. With external quiescence as input, these attractors represent the long-term state of normal HSCs where genes related to metabolism or cell growth are repressed (e.g., *MYC*, *SOX4*, *MAPK1*, *MTOR*), and cell cycle inhibitors are activated (e.g., *CDKN* genes, *RB1*). Furthermore, the merged model provides a more comprehensive view of gene regulation. Both original models only capture part of the regulations of the *TP53* tumor suppressor gene: the Palma model states that *ARF* (*CDKN2A*) activates *p53* (*TP53*); and the Ikonomi model describes the negative feedback of *MDM2*. Integrating the models provides a broader view of *p53* regulation, where *ARF* activates *p53* through the inhibition of *MDM2* [58].

An important modeling goal is to predict clinical outcomes for patients with specific mutations. In order to connect GRN with phenotypic manifestations of disease, phenotype nodes are introduced to represent high-level cellular behaviors such as *apoptosis*, *proliferation*, and *differentiation*. These nodes are defined as logical or arithmetic functions of key molecular regulators. Palma et al. have also defined an integrated network score as a proxy for patients’ clinical outcomes, which is calculated by subtracting the values of *apoptosis* and *differentiation* from the value of the *proliferation* [7]. Using this metric, we were able to analyze the model’s performance on predicting (1) the impact of mutations on overall survival, as measured by the Cox hazard ratio from the AMLSG dataset [23]; and (2) the progression of disease, as measured by the percentage of blast cells from the TCGA dataset [17]. The Cox hazard ratio quantifies how the presence of a particular mutation influences patient survival, with higher values indicating increased risk of death. The percentage of blast cells in the bone marrow or peripheral blood, on the other hand, is a key diagnostic and prognostic indicator in AML, with higher percentages associated with more aggressive disease and poorer prognosis. Replicating the results of Palma, we found that our merged model effectively captures the clinical implications of the genetic mutations, with high correlation between the simulated scores from patients with gene mutations of *FLT3*, *NPM1* and *DNMT3A,* and the measured blast percentage (Table S3). Further, we applied the model to an independent large AML dataset, Beat AML [20]. Both the Palma model (Fig. 3C) and merged model (Fig. 3D) show robustness of predicting disease progression on similar tasks*.*

A limitation of any model with a limited number of genes is that patients may have mutations in other genes that also play important roles in AML prognosis. Our goal with model merging is to capture a more comprehensive genetic landscape, and improve the performance of prediction for a wider range of patients. Therefore, we expanded the patients’ mutation profiles to all available genes covered by the merged model. The performance of the Palma model declined when expanding the coverage, represented by the lower correlation between the model network score and clinical measurement of the blast percentage (Fig. 3E), while the merged model improved the prediction power significantly (Fig. 3F). This can be attributed to the richer information captured in the extended model. For example, clinical responses of the *TP53*-mutated patients can be modeled more accurately using the merged model, as shown by the light green circles. This is not surprising, as the Palma model does not include the detailed interactions around TP53, whereas this is included in the Ikonomi paper.

## Discussion

The study provides a semi-automatic approach to merge GRN models based on logic rules. One significant advantage for merging GRN models is to increase the coverage of patients with different mutations, thereby enhancing the model’s applicability. For example, *NRAS* is mutated in 13.7% of the AML patients from the Beat AML dataset, but is not modelled by Palma et al. The potential impact of *NRAS* on disease progression can be described with improved accuracy using the merged Palma-Ikonomi model (Fig. 3F). Similarly, merging the Bonzanni model with the Krumsiek model expands the coverage of the 5.3% patients who have *CEBPA* mutations. By including more genes in the models, they can better reflect the genetic diversity observed in AML patients.

Pioneering efforts such as CNORfeeder [59] extend edge-level prior-knowledge networks (PKNs) by adding links mined from perturbation data. More recently, single-cell GRN studies have proposed integrating interaction graphs inferred from different cell states [60]. These approaches first enlarge a prior-knowledge network and then try to find regulatory relationships between them, but they do not tackle the problem of merging two or more pre-existing executable rule sets while preserving their validated dynamics. LM-Merger operates one layer downstream, where it (i) harmonizes identifiers, (ii) resolves rule conflicts, and (iii) verifies that emergent attractors and predictive performance are preserved or improved. While we build on libSBML [61] for low-level XML handling, libSBML alone cannot carry out any of these capabilities. The resulting integrated logical model therefore complements, rather than replaces, PKN-expansion frameworks. Once an enriched edge list has been converted to a model with explicit logic, LM-Merger can further reconcile it with additional executable models.

There are different rules that can be defined for the merged models. In our current workflow, users can select one of the three deterministic methods—AND, OR, or Inhibitor Wins—for the integration. While this provides a structured approach, it would be advantageous to establish a biologically driven rationale for choosing among these three methods. Furthermore, there may be instances where a single method applied across the entire network is insufficient. For example, a user might choose to apply the Inhibitor Wins method to nodes where inhibitory interactions dominate, such as genes with well-established repressive regulators, while using the AND method for nodes where activation requires strict synergy between inputs. As an alternative, one could also consider a probabilistic approach, where probabilities are assigned to each logical rule from the individual models [62, 63]. In biology, gene expression and TF binding have a stochastic nature, which is reflected in the random and context-dependent interactions among biological entities [64, 65]. Therefore, as a potential next step, we may consider implementing a probabilistic approach that can better model the uncertainty and variability in regulatory interactions. In general, researchers will have to carefully consider how to merge rules from different models, depending on their research objectives, biological contexts, and the performance of the models.

Beyond Boolean rules, some networks use multi-valued or probabilistic logic. Before merging such models, node states should first be harmonized—either by binarizing discrete levels or by rescaling them onto a shared categorical scale—to ensure compatible truth tables. For probabilistic models, rule weights (e.g. activation probabilities or confidence scores) can be combined through weighted logic, so that the merged network can retain stochasticity and to probe robustness under simulated noise. LM-Merger already accepts “booleanised” versions of multi-valued models, and full multi-valued and probabilistic support is under active development.

The application of this model merging workflow relies on access to existing models. Although repositories for logical models exist, they are primarily for internal validation and are not easily mapped to other external models [66]. Moreover, extensive annotation is required to determine the biological relevance of the models, including their knowledge sources and the overall purpose of the model. Automated model annotation is promising to advance the understanding and reuse of biosimulation models. Therefore, we are currently working on extending our previously built tool for quantitative SBML models [67] to qualitative models.

Our workflow also depends on the reproducibility of models. In our study, we evaluated the reproducibility of each individual model before merging, and then compared the performance of the merged models with the original models across datasets. In the four models we considered, we were able to reproduce their performance (except for a minor discrepancy in the Palma model, which we corrected by contacting the authors). Our results highlight the importance of high-quality models for the merging workflow to succeed. We are actively collaborating with the logical modeling community to develop guidelines and automated tools that support reproducibility, including standardization and annotation of qualitative models [68]. With a repository of well-annotated logical GRN models, our workflow can be more readily applied, thereby offering greater value to the research community.

Different simulation processes, e.g., updating schemas, or simulation tools and platforms, can yield varying outcomes. In our study, we evaluated both synchronous and asynchronous updating strategies across two model pairs, and while asynchronous updating identified more cyclic attractors, the overall conclusions remained consistent between both approaches. Our workflow does not impose specific requirements on the simulation process, allowing researchers to explore model behavior under different circumstances and select the one most appropriate for their study. Logical models should ideally be tool-agnostic, provided they follow standardized formats such as SBML-qual, allowing for simulation testing across different environments.

In the longer term, we view model merging as an important part of biomedical digital twins research [69]. A digital twin is a set of virtual information constructs that mimics the structure, context, and behavior of the physical twin, and is dynamically updated with data from its physical twin [70]. The model merging workflow allows researchers to systematically construct a virtual representation of a disease by integrating previously developed models. We argue that the merged models provide candidate models for biomedical digital twins; Ideally, a large merged model could be combined with pertinent patient data, to generate patient-specific models for predicting response to personalized treatments. In the case of AML, model personalization is possible by integrating mutation states of patients and drug response data available in datasets such as Beat AML. These personalized models could be used to test “in silico” for individual responses to drug interventions.

## Conclusions

In summary, we demonstrated the effectiveness of the workflow by merging two pairs of AML-focused models, capturing the complex interactions and regulatory mechanisms involved in gene expression and clinical outcomes. The merged models align well with biological phenomena and provide robust predictions, as evidenced by the strong correlations with experimental and clinical data. Our work also underscores the importance of standardization, reproducibility, and systematic documentation in logical models, and calls for comprehensive, high-quality, annotated data. By addressing these challenges, the workflow has the potential to advance our understanding of complex biological systems and support the development of more effective and personalized therapies for diseases like AML.

## Electronic supplementary material

Below is the link to the electronic supplementary material.

Supplementary Material 1

## Data Availability

The workflow and accompanying tools, including modules for model standardization, automated logical model merging, and evaluation, are available at https://github.com/IlyaLab/LogicModelMerger/. Models are collected from their publications. Data used to test the models are all obtained from public repositories or publications, for details, please see Data section in the Supplementary file.

## Abbreviations

GRN: gene regulatory network
AML: acute myeloid leukemia
WCM: whole-cell models
CALM: curation and annotation of logical models initiative
SBML: Systems Biology Markup Language
SBML-qual: Systems Biology Markup Language Qualitative Models
HGNC: the HUGO Gene Nomenclature Committee
TF: transcription factor
HSC: hematopoietic stem cells

## References

[1] Karlebach G, Shamir R. Modelling and analysis of gene regulatory networks. Nat Rev Mol Cell Biol. 2008;9(10):770–80. 18797474 10.1038/nrm2503

[2] Le Novère N. Quantitative and logic modelling of molecular and gene networks. Nat Rev Genet. 2015;16(3):146–58. 25645874 10.1038/nrg3885 PMC4604653

[3] Rothenberg EV. Causal gene regulatory network modeling and genomics: second-generation challenges. J Comput Biol. 2019;26(7):703–18. 31063008 10.1089/cmb.2019.0098 PMC6661971

[4] Abou-Jaoudé W, Traynard P, Monteiro PT, Saez-Rodriguez J, Helikar T, Thieffry D, et al. Logical modeling and dynamical analysis of cellular networks. Front Genet. 2016;31(7):94. 10.3389/fgene.2016.00094 PMC4885885 27303434

[5] Sgariglia D, et al. Optimizing therapeutic targets for breast cancer using boolean network models. Comput Biol Chem. 2024;109:108022. 38350182 10.1016/j.compbiolchem.2024.108022

[6] Plaugher D, Aguilar B, Murrugarra D. Uncovering potential interventions for pancreatic cancer patients via mathematical modeling. J Theor Biol. 2022;7(548): 111197. 10.1016/j.jtbi.2022.111197 35752283

[7] Palma A, Iannuccelli M, Rozzo I, Licata L, Perfetto L, Massacci G, et al. Integrating patient-specific information into logic models of complex diseases: application to acute myeloid Leukemia. J Pers Med. 2021;11(2):117. 33578936 10.3390/jpm11020117 PMC7916657

[8] Levine M, Davidson EH. Gene regulatory networks for development. Proc Natl Acad Sci. 2005;102(14):4936–42. 15788537 10.1073/pnas.0408031102 PMC555974

[9] Davidson EH. Emerging properties of animal gene regulatory networks. Nature. 2010;468(7326):911–20. 21164479 10.1038/nature09645 PMC3967874

[10] Agmon E, Spangler RK, Skalnik CJ, Poole W, Peirce SM, Morrison JH, et al. Vivarium: an interface and engine for integrative multiscale modeling in computational biology. Bioinformatics. 2022;38(7):1972–9. 35134830 10.1093/bioinformatics/btac049 PMC8963310

[11] Liu ZP, Wu C, Miao H, Wu H. RegNetwork: an integrated database of transcriptional and post-transcriptional regulatory networks in human and mouse. Database J Biol Databases Curation. 2015;2015:095. 10.1093/database/bav095 PMC4589691 26424082

[12] Pillich RT, Chen J, Churas C, Fong D, Gyori BM, Ideker T, et al. NDEx IQuery: a multi-method network gene set analysis leveraging the Network Data Exchange. Bioinformatics. 2023;39(3):btad118. 36882166 10.1093/bioinformatics/btad118 PMC10023220

[13] Karr JR, Takahashi K, Funahashi A. The principles of whole-cell modeling. Curr Opin Microbiol. 2015;27:18–24. 26115539 10.1016/j.mib.2015.06.004

[14] Karr JR, Sanghvi JC, Macklin DN, Gutschow MV, Jacobs JM, Bolival B, et al. A whole-cell computational model predicts phenotype from genotype. Cell. 2012;150(2):389–401. 22817898 10.1016/j.cell.2012.05.044 PMC3413483

[15] Macklin DN, Ahn-Horst TA, Choi H, Ruggero NA, Carrera J, Mason JC, et al. Simultaneous cross-evaluation of heterogeneous *E. coli* datasets via mechanistic simulation. Science. 2020;369(6502):eaav3751. 32703847 10.1126/science.aav3751 PMC7990026

[16] Georgouli K, Yeom JS, Blake RC, Navid A. Multi-scale models of whole cells: progress and challenges. Front Cell Dev Biol. 2023;7(11):1260507. 10.3389/fcell.2023.1260507 PMC10661945 38020904

[17] Cancer Genome Atlas Research Network, Ley TJ, Miller C, Ding L, Raphael BJ, Mungall AJ, et al. Genomic and epigenomic landscapes of adult de novo acute myeloid leukemia. N Engl J Med. 2013;368(22):2059–74. 23634996 10.1056/NEJMoa1301689 PMC3767041

[18] Siveen KS, Uddin S, Mohammad RM. Targeting acute myeloid leukemia stem cell signaling by natural products. Mol Cancer. 2017;16(1):13. 28137265 10.1186/s12943-016-0571-x PMC5282735

[19] Tyner JW, Tognon CE, Bottomly D, Wilmot B, Kurtz SE, Savage SL, et al. Functional genomic landscape of acute myeloid leukaemia. Nature. 2018;562(7728):526–31. 30333627 10.1038/s41586-018-0623-z PMC6280667

[20] Bottomly D, Long N, Schultz AR, Kurtz SE, Tognon CE, Johnson K, et al. Integrative analysis of drug response and clinical outcome in acute myeloid leukemia. Cancer Cell. 2022;40(8):850-864.e9. 35868306 10.1016/j.ccell.2022.07.002 PMC9378589

[21] Malani D, Kumar A, Brück O, Kontro M, Yadav B, Hellesøy M, et al. Implementing a functional precision medicine tumor board for acute myeloid Leukemia. Cancer Discov. 2022;12(2):388–401. 34789538 10.1158/2159-8290.CD-21-0410 PMC9762335

[22] Qin G, Dai J, Chien S, Martins TJ, Loera B, Nguyen QH, et al. Mutation patterns predict drug sensitivity in acute myeloid Leukemia. Clin Cancer Res Off J Am Assoc Cancer Res. 2024;30(12):2659–71. 10.1158/1078-0432.CCR-23-1674 PMC11176916 38619278

[23] Papaemmanuil E, Gerstung M, Bullinger L, Gaidzik VI, Paschka P, Roberts ND, et al. Genomic classification and prognosis in acute myeloid Leukemia. N Engl J Med. 2016;374(23):2209–21. 27276561 10.1056/NEJMoa1516192 PMC4979995

[24] Hérault L, Poplineau M, Duprez E, Remy É. A novel Boolean network inference strategy to model early hematopoiesis aging. Comput Struct Biotechnol J. 2023;21:21–33. 36514338 10.1016/j.csbj.2022.10.040 PMC9719905

[25] Silverbush D, Grosskurth S, Wang D, Powell F, Gottgens B, Dry J, et al. Cell-specific computational modeling of the PIM pathway in acute myeloid Leukemia. Cancer Res. 2017;77(4):827–38. 27965317 10.1158/0008-5472.CAN-16-1578

[26] Helikar T, Kowal B, McClenathan S, Bruckner M, Rowley T, Madrahimov A, et al. The cell collective: toward an open and collaborative approach to systems biology. BMC Syst Biol. 2012;7(6):96. 10.1186/1752-0509-6-96 PMC3443426 22871178

[27] Naldi A, Hernandez C, Abou-Jaoudé W, Monteiro PT, Chaouiya C, Thieffry D. Logical modeling and analysis of cellular regulatory networks with GINsim 3.0. Front Physiol [Internet]. 2018;9:1. 10.3389/fphys.2018.00646/full. 29971008 10.3389/fphys.2018.00646 PMC6018412

[28] sybila/NewBioDiVinE [Internet]. sybila; 2014 [cited 2024 Jul 8]. Available from: https://github.com/sybila/NewBioDiVinE

[29] Kanehisa M, Goto S. KEGG: kyoto encyclopedia of genes and genomes. Nucl Acids Res. 2000;28(1):27–30. 10592173 10.1093/nar/28.1.27 PMC102409

[30] Reactome Pathway Knowledgebase 2024| Nucleic Acids Research| Oxford Academic [Internet]. [cited 2024 May 21]. Available from: https://academic.oup.com/nar/article/52/D1/D672/7369850?login=false&utm_source=advanceaccess&utm_campaign=nar&utm_medium=email

[31] Lo Surdo P, Iannuccelli M, Contino S, Castagnoli L, Licata L, Cesareni G, et al. SIGNOR 3.0, the SIGnaling network open resource 3.0: 2022 update. Nucl Acids Res. 2022;51(D1):D631–7. 10.1093/nar/gkac883 PMC9825604 36243968

[32] Shin J, Porubsky V, Carothers J, Sauro HM. Standards, dissemination, and best practices in systems biology. Curr Opin Biotechnol. 2023;1(81): 102922. 10.1016/j.copbio.2023.102922 PMC10435326 37004298

[33] Niarakis A, Kuiper M, Ostaszewski M, Malik Sheriff RS, Casals-Casas C, Thieffry D, et al. Setting the basis of best practices and standards for curation and annotation of logical models in biology—highlights of the [BC]2 2019 CoLoMoTo/SysMod Workshop. Brief Bioinform. 2021;22(2):1848–59. 32313939 10.1093/bib/bbaa046 PMC7986594

[34] Chaouiya C, Bérenguier D, Keating SM, Naldi A, van Iersel MP, Rodriguez N, et al. SBML qualitative models: a model representation format and infrastructure to foster interactions between qualitative modelling formalisms and tools. BMC Syst Biol. 2013;10(7):135. 10.1186/1752-0509-7-135 PMC3892043 24321545

[35] Chaouiya C, Keating SM, Berenguier D, Naldi A, Thieffry D, van Iersel MP, et al. The systems biology Markup language (SBML) level 3 package: qualitative models, version 1, release 1. J Integr Bioinforma. 2015;12(2):270. 10.2390/biecoll-jib-2015-270 26528568

[36] Seal RL, Braschi B, Gray K, Jones TEM, Tweedie S, Haim-Vilmovsky L, et al. Genenames.org: the HGNC resources in 2023. Nucl Acids Res. 2023;51(D1):D1003–9. 36243972 10.1093/nar/gkac888 PMC9825485

[37] International Protein Nomenclature Guidelines [Internet]. [cited 2024 Jun 4]. Available from: https://www.ncbi.nlm.nih.gov/genbank/internatprot_nomenguide/

[38] Porubsky VL, Sauro HM. A practical guide to reproducible modeling for biochemical networks. In: Nguyen LK, editor. Computational modeling of signaling networks. New York: Springer; 2023. p. 107–38. 10.1007/978-1-0716-3008-2_5. 10.1007/978-1-0716-3008-2_5 37074576

[39] Blinov ML, Gennari JH, Karr JR, Moraru II, Nickerson DP, Sauro HM. Practical resources for enhancing the reproducibility of mechanistic modeling in systems biology. Curr Opin Syst Biol. 2021;1(27): 100350.

[40] Naldi A, Hernandez C, Levy N, Stoll G, Monteiro PT, Chaouiya C, et al. The CoLoMoTo interactive notebook: accessible and reproducible computational analyses for qualitative biological networks. Front Physiol. 2018;19(9):680. 10.3389/fphys.2018.00680 PMC6018415 29971009

[41] Thomas R, D’Ari R. Biological Feedback. CRC Press; 1990. 328 p.

[42] Deng X, Chen Y. Inference of gene regulations between multiple activators/inhibitors and singular genes. In: 2018 9th international conference on information technology in medicine and education (ITME) [Internet]; 2018. p. 192–8. Available from: https://ieeexplore.ieee.org/document/8589283

[43] Encyclopedia of Bioinformatics and Computational Biology: ABC of Bioinformatics. Elsevier; 2018. 3421 p.

[44] Lambert SA, Jolma A, Campitelli LF, Das PK, Yin Y, Albu M, et al. The human transcription factors. Cell. 2018;172(4):650–65. 29425488 10.1016/j.cell.2018.01.029 PMC12908702

[45] Reiter F, Wienerroither S, Stark A. Combinatorial function of transcription factors and cofactors. Curr Opin Genet Dev. 2017;1(43):73–81. 10.1016/j.gde.2016.12.007 28110180

[46] Reynolds N, O’Shaughnessy A, Hendrich B. Transcriptional repressors: multifaceted regulators of gene expression. Development. 2013;140(3):505–12. 23293282 10.1242/dev.083105

[47] Samaga R, Klamt S. Modeling approaches for qualitative and semi-quantitative analysis of cellular signaling networks. Cell Commun Signal. 2013;11(1):43. 23803171 10.1186/1478-811X-11-43 PMC3698152

[48] Long NA, Golla U, Sharma A, Claxton DF. Acute myeloid Leukemia stem cells: origin, characteristics, and clinical implications. Stem Cell Rev Rep. 2022;18(4):1211–26. 35050458 10.1007/s12015-021-10308-6 PMC10942736

[49] Bonzanni N, Garg A, Feenstra KA, Schütte J, Kinston S, Miranda-Saavedra D, et al. Hard-wired heterogeneity in blood stem cells revealed using a dynamic regulatory network model. Bioinforma Oxf Engl. 2013;29(13):i80-88. 10.1093/bioinformatics/btt243 PMC3694641 23813012

[50] Krumsiek J, Marr C, Schroeder T, Theis FJ. Hierarchical differentiation of myeloid progenitors is encoded in the transcription factor network. PLoS ONE. 2011;6(8): e22649. 21853041 10.1371/journal.pone.0022649 PMC3154193

[51] Jalili M, Yaghmaie M, Ahmadvand M, Alimoghaddam K, Mousavi SA, Vaezi M, et al. Prognostic value of RUNX1 mutations in AML: a meta-analysis. Asian Pac J Cancer Prev APJCP. 2018;19(2):325–9. 29479958 10.22034/APJCP.2018.19.2.325 PMC5980915

[52] Su L, Shi YY, Liu ZY, Gao SJ. Acute myeloid Leukemia with CEBPA mutations: Current progress and future directions. Front Oncol. 2022;1(12): 806137. 10.3389/fonc.2022.806137 PMC8844020 35178345

[53] Chambers SM, Boles NC, Lin KYK, Tierney MP, Bowman TV, Bradfute SB, et al. Hematopoietic fingerprints: an expression database of stem cells and their progeny. Cell Stem Cell. 2007;1(5):578–91. 18371395 10.1016/j.stem.2007.10.003 PMC2475548

[54] Knudsen KJ, Rehn M, Hasemann MS, Rapin N, Bagger FO, Ohlsson E, et al. ERG promotes the maintenance of hematopoietic stem cells by restricting their differentiation. Genes Dev. 2015;29(18):1915–29. 26385962 10.1101/gad.268409.115 PMC4579349

[55] Takai J, Moriguchi T, Suzuki M, Yu L, Ohneda K, Yamamoto M. The Gata1 5′ region harbors distinct cis-regulatory modules that direct gene activation in erythroid cells and gene inactivation in HSCs. Blood. 2013;122(20):3450–60. 24021675 10.1182/blood-2013-01-476911

[56] Ramos CA, Bowman TA, Boles NC, Merchant AA, Zheng Y, Parra I, et al. Evidence for diversity in transcriptional profiles of single hematopoietic stem cells. PLoS Genet. 2006;2(9): e159. 17009876 10.1371/journal.pgen.0020159 PMC1584276

[57] Ikonomi N, Kühlwein SD, Schwab JD, Kestler HA. Awakening the HSC: dynamic modeling of HSC maintenance unravels regulation of the TP53 pathway and quiescence. Front Physiol. 2020;11:848. 32848827 10.3389/fphys.2020.00848 PMC7411231

[58] Sherr CJ, Weber JD. The ARF/p53 pathway. Curr Opin Genet Dev. 2000;10(1):94–9. 10679383 10.1016/s0959-437x(99)00038-6

[59] Eduati F, De Las RJ, Di Camillo B, Toffolo G, Saez-Rodriguez J. Integrating literature-constrained and data-driven inference of signalling networks. Bioinformatics. 2012;28(18):2311–7. 22734019 10.1093/bioinformatics/bts363 PMC3436796

[60] Stock M, Losert C, Zambon M, Popp N, Lubatti G, Hörmanseder E, et al. Leveraging prior knowledge to infer gene regulatory networks from single-cell RNA-sequencing data. Mol Syst Biol. 2025;21(3):214–30. 39939367 10.1038/s44320-025-00088-3 PMC11876610

[61] Bornstein BJ, Keating SM, Jouraku A, Hucka M. LibSBML: an API library for SBML. Bioinformatics. 2008;24(6):880–1. 18252737 10.1093/bioinformatics/btn051 PMC2517632

[62] Shmulevich I, Dougherty ER, Kim S, Zhang W. Probabilistic Boolean networks: a rule-based uncertainty model for gene regulatory networks. Bioinformatics. 2002;18(2):261–74. 11847074 10.1093/bioinformatics/18.2.261

[63] Trairatphisan P, Mizera A, Pang J, Tantar A, Schneider J, Sauter T. Recent development and biomedical applications of probabilistic Boolean networks. Cell Commun Signal. 2013;11(1):46. 23815817 10.1186/1478-811X-11-46 PMC3726340

[64] McAdams HH, Arkin A. It’s a noisy business! Genetic regulation at the nanomolar scale. Trends Genet TIG. 1999;15(2):65–9. 10098409 10.1016/s0168-9525(98)01659-x

[65] Parab L, Pal S, Dhar R. Transcription factor binding process is the primary driver of noise in gene expression. PLOS Genet. 2022;18(12): e1010535. 36508455 10.1371/journal.pgen.1010535 PMC9779669

[66] Malik-Sheriff RS, Glont M, Nguyen TVN, Tiwari K, Roberts MG, Xavier A, et al. BioModels—15 years of sharing computational models in life science. Nucl Acids Res. 2020;48(D1):D407–15. 31701150 10.1093/nar/gkz1055 PMC7145643

[67] Shin W, Gennari JH, Hellerstein JL, Sauro HM. An automated model annotation system (AMAS) for SBML models. Bioinformatics. 2023;39(11):btad658. 37882737 10.1093/bioinformatics/btad658 PMC10628433

[68] Niarakis A, Waltemath D, Glazier J, Schreiber F, Keating SM, Nickerson D, et al. Addressing barriers in comprehensiveness, accessibility, reusability, interoperability and reproducibility of computational models in systems biology. Brief Bioinform. 2022;23(4):bbac212. 35671510 10.1093/bib/bbac212 PMC9294410

[69] Hernandez-Boussard T, Macklin P, Greenspan EJ, Gryshuk AL, Stahlberg E, Syeda-Mahmood T, et al. Digital twins for predictive oncology will be a paradigm shift for precision cancer care. Nat Med. 2021;27(12):2065–6. 34824458 10.1038/s41591-021-01558-5 PMC9097784

[70] Foundational Research Gaps and Future Directions for Digital Twins [Internet]. Washington, D.C.: National Academies Press; 2024 [cited 2024 Aug 26]. Available from: https://www.nap.edu/catalog/26894 39088664

[71] Longabaugh WJ, Davidson EH, Bolouri H. Visualization, documentation, analysis, and communication of large-scale gene regulatory networks. Biochimica et Biophysica Acta (BBA)-Gene Regulatory Mechanisms. 2009;1789(4):363-74. 18757046 10.1016/j.bbagrm.2008.07.014 PMC2762351

